# Auxin flow-mediated competition between axillary buds to restore apical dominance

**DOI:** 10.1038/srep35955

**Published:** 2016-11-08

**Authors:** Jozef Balla, Zuzana Medveďová, Petr Kalousek, Natálie Matiješčuková, Jiří Friml, Vilém Reinöhl, Stanislav Procházka

**Affiliations:** 1 CEITEC-Central European Institute of Technology, Mendel University in Brno, Zemedelska 1, 613 00 Brno, Czech Republic; 2 Department of Plant Biology, Mendel University in Brno, Zemedelska 1, 613 00 Brno, Czech Republic; 3 Department of Molecular Biology and Radiobiology, Mendel University in Brno, Zemedelska 1, 613 00 Brno, Czech Republic; 4 Institute of Science and Technology Austria (IST Austria), 3400 Klosterneuburg, Austria

## Abstract

Apical dominance is one of the fundamental developmental phenomena in plant biology, which determines the overall architecture of aerial plant parts. Here we show apex decapitation activated competition for dominance in adjacent upper and lower axillary buds. A two-nodal-bud pea (*Pisum sativum* L.) was used as a model system to monitor and assess auxin flow, auxin transport channels, and dormancy and initiation status of axillary buds. Auxin flow was manipulated by lateral stem wounds or chemically by auxin efflux inhibitors 2,3,5-triiodobenzoic acid (TIBA), 1-*N*-naphtylphtalamic acid (NPA), or protein synthesis inhibitor cycloheximide (CHX) treatments, which served to interfere with axillary bud competition. Redirecting auxin flow to different points influenced which bud formed the outgrowing and dominant shoot. The obtained results proved that competition between upper and lower axillary buds as secondary auxin sources is based on the same auxin canalization principle that operates between the shoot apex and axillary bud.

It is widely accepted that genetic determination and variable responses to different environmental conditions are responsible for the wide range of plant body forms. Among other factors, plasticity in plant shape is possible due to activity of the shoot apical meristem (SAM), which is established during embryogenesis. The entire aboveground part of the plant mass originates from this single primary meristem on the upper end of plant axis. Primary SAM continually produces new stem cells, leaf primordia, which develop into leaves. In each new immature leaf axil, secondary SAMs (or axillary meristems) are forming. If these meristems are arrested, dormant axillary buds form, which contain immature leaves and tertiary axillary meristems. These buds can be activated by large-scale environmental stimuli, such as shading, temperature, water, and nutrient availability; and loss of SAM by wind damage or grazing by herbivorous animals, which results in secondary branch production. Plants build primary, secondary and/or tertiary bodies in a modular fashion, adding new to existing axes in this hierarchical order based on environmental conditions (reviewed in ref. 1).

Apical dominance is the regulatory mechanism which primary SAM uses to control outgrowth of higher order axillary meristems. Simple primary SAM removal releases one or more axillary buds from dormancy and the outgrowing shoots replace the previously dominant apex. Auxin is the most studied signalling molecule originating in SAM. Auxin is synthesized in the shoot apex and young leaves, and transported in stem vasculature, i.e., downwards in xylem parenchyma cells. The direction of basipetal cell-to-cell auxin transport is determined by polar, subcellular localization of PIN auxin efflux carriers^2^,^3^. These carriers occur at the lower side of the respective cells assisted by AUX1/LAX auxin influx carriers, which also show polar localization in some cells^4^. This tip-to-base directed auxin flow maintains dormancy in axillary buds. Indeed, external auxin supply into decapitated stem prevents bud outgrowth^5^. On contrary, auxin application to axillary buds of decapitated plants does not block their outgrowth, suggesting the bud outgrowth inhibition mechanism(s) is not simply auxin supply from stem to buds confirmed by application of radioactively labelled auxin to a stump, which did not enter the axillary buds^6^. In addition, it was showed^7^ that arrested axillary buds promptly increased auxin production following activation.

One of the possibilities how to explain this behaviour of auxin is the competitive canalization model^8^,^9^,^10^,^11^. The original canalization hypothesis proposed an initial broad and low auxin flux from a source, which up-regulated and directed its own transport. The transport finally narrowed to cell files, canals, where the hormone moved very effectively from source to sink. These auxin transport channels subsequently patterned a new plant vasculature^12^. This concept was applied to bud outgrowth regulation: forming effective auxin transport channels from dormant axillary buds to the primary stem auxin flow was a prerequisite for its outgrowth. However, this in the presence of a strong auxin source–the primary SAM that supplied auxin to the primary stem and reduced its sink strength for possible secondary auxin sources–was disabled^12^.

Aim of the present study was to show that not only the primary stem apex and axillary buds, but also adjacent axillary buds compete for dominance. We also tested the importance of long-range auxin signalling mediating bud outgrowth.

## Results

### Axillary buds released from dormancy compete for dominance in pea

**Figure 1:**
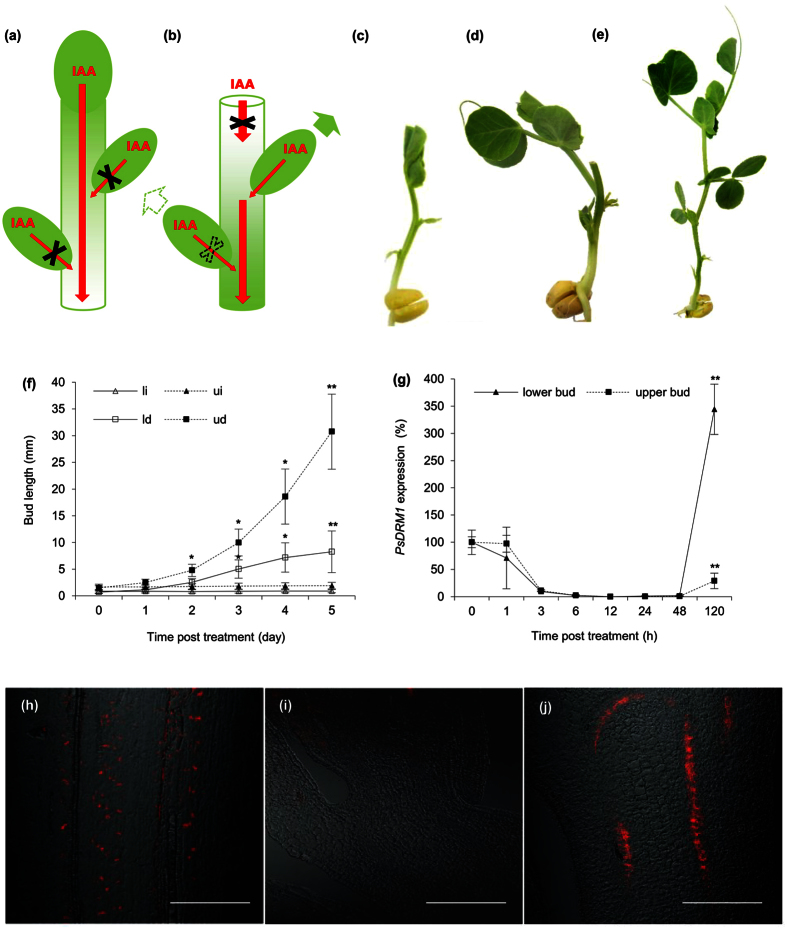
Axillary buds released from dormancy compete for dominance in pea. **(a)** Scheme of intact plant. Red arrows represent auxin (IAA) flow; red arrows crossed with black X represent disabled auxin flow. Auxin loaded from the apex (as primary source) to the stem prevents auxin canalization and its export from the axillary buds (as potential secondary auxin sources). **(b)** Scheme of decapitated plant. Red and crossed red arrows as depicted in a). Dashed crossed red arrow represents intermitted auxin flow after temporary activation. Green arrow represents bud outgrowth and dominance, dashed green arrow represents temporary outgrowth. Apex, the primary source for auxin flow, is removed and auxin synthesized in the buds can be exported, resulting in outgrowth of both buds. The initial outgrowth turns into competition leading to upper bud dominance over the lower. **(c)** Intact control plant 7-DAS (at the beginning of experiment). **(d)** Plant 5 days after decapitation with outgrowing and dominant upper axillary bud and temporarily outgrown and then arrested lower bud. **(e)** Intact plant of same age (7-DAS + 5 days); both axillary buds remain arrested. **(f)** Length of axillary buds and forming shoots, where: (li) lower bud of intact plants; (ui) upper bud of intact plants; (ld) lower bud of decapitated plants; (ud) upper bud of decapitated plants. Statistically significant differences (identified by Student’s t-test): α = 0.05* and α = 0.01**. Error bars represent standard deviations (n = 60). **(g)** Relative expression of *PsDRM1* gene in lower and upper axillary buds following decapitation. Statistically significant differences (identified by Student’s t-test): α = 0.05* and α = 0.01**. Error bars represent standard deviations (n = 4). **(h,j)** Immunoanalysis of PIN1 auxin efflux carriers (red signal) showed polar localization in the primary stem **(h)**, lack of localization in procambial cells of inhibited axillary buds, **(i)** and polar localization in procambial cells of outgrowing buds **(j)**. Scale bar, 100 μm.

Garden pea, among plant species, has well pronounced apical dominance. This trait manifests by inhibition of axillary bud outgrowth by the shoot apex (Fig. 1a,c,e). In the primary stem of intact pea plants, PIN1 auxin efflux carriers in cells accompanying the vasculature were polarly organized, indicating massive polar auxin transport (PAT) (Fig. 1h). On contrary, PIN1 carrier localization in procambial cell files of inhibited axillary buds did not show this polarized distribution (Fig. 1i). Shoot apex removal releases axillary buds from growth inhibition (Fig. 1b,d) and in these released outgrowing buds the PIN1 carriers in procambial cells became polarly localized (Fig. 1j), similar to the primary stem. In our two-nodal-bud model system the lower axillary buds were half the length of the upper buds, however following decapitation, bud growth was initiated and occurred at the same rate, i.e., bud length doubled daily. Three days after decapitation, growth rates began to slow in shoots that developed from lower buds, while upper buds continued growth at the same rate and became evidently dominant (Fig. 1f). This competition pattern for outgrowth of axillary buds was not altered when cotyledons, the nutrient sources, were removed (Supplementary Fig. 1a). Morphological changes in buds were accompanied by expected changes in the dormancy marker gene *PsDRM1* (*DORMANCY-ASSOCIATED PROTEIN1*) expression, reflecting bud growth status, i.e., in both outgrowing buds, *PsDRM1* expression decreased to zero during six hours after decapitation, while in rearrested lower buds at day five, expression increased to a high level (Fig. 1g).

### Auxin pool in decapitated stem delays release of buds from dormancy

**Figure 2:**
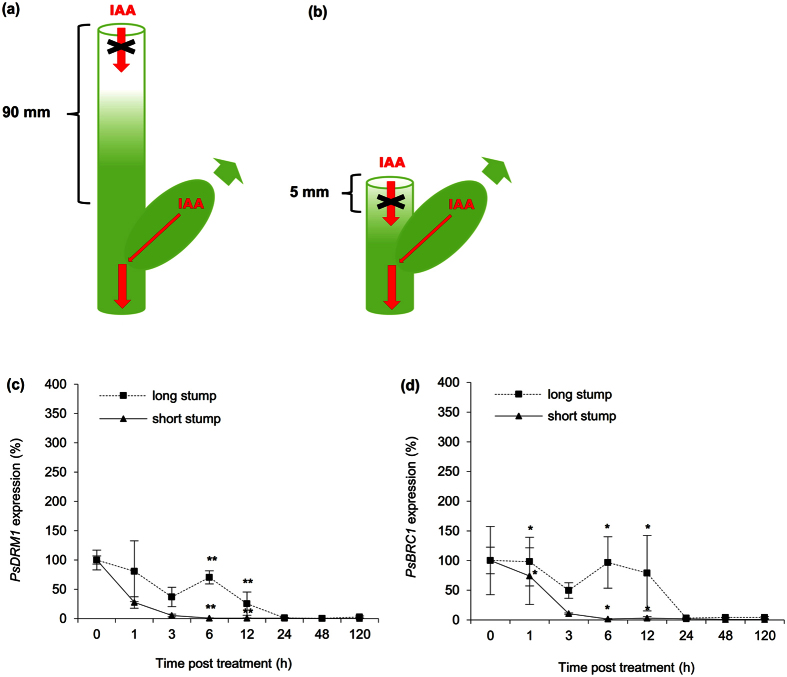
Auxin pool in decapitated stem delays release of buds from dormancy. **(a,b)** Scheme of plant with long stump decapitated 90 mm above the upper bud **(a)** and plant with short stump decapitated 5 mm above the upper bud **(b)**. Red arrows represent auxin (IAA) flow; red arrows crossed with black X represent disabled auxin flow. Green arrows represent bud outgrowth. Auxin depletion or decrease from the missing apex has impact on bud outgrowth timing. **(c,d)** Relative expression of *PsDRM1* **(c)** and *PsBRC1* **(d)** genes in the upper axillary bud of plants with long or short stump. Statistically significant differences (identified by Student’s t-test): α = 0.05* and α = 0.01**. Error bars represent standard deviations (n = 4).

The importance of basipetal auxin flow in stems for bud outgrowth regulation was evaluated using de-etiolated plants with long internodes. Plants with the decapitation site and upper axillary bud separated by 90 mm (long stump) were compared with the standard 5 mm (short stump) separation (Fig. 2a,b). Dormancy release and bud outgrowth timing were determined using the dormancy marker gene *PsDRM1* and branching repressor gene *PsBRC1* (*BRANCHED1*). Generally, we observed an approximately 12 h delay in the expression dynamics of both genes in long compared to short stem stump plants (Fig. 2c,d).

### Interruption of PAT in the primary stem releases buds from dormancy

**Figure 3:**
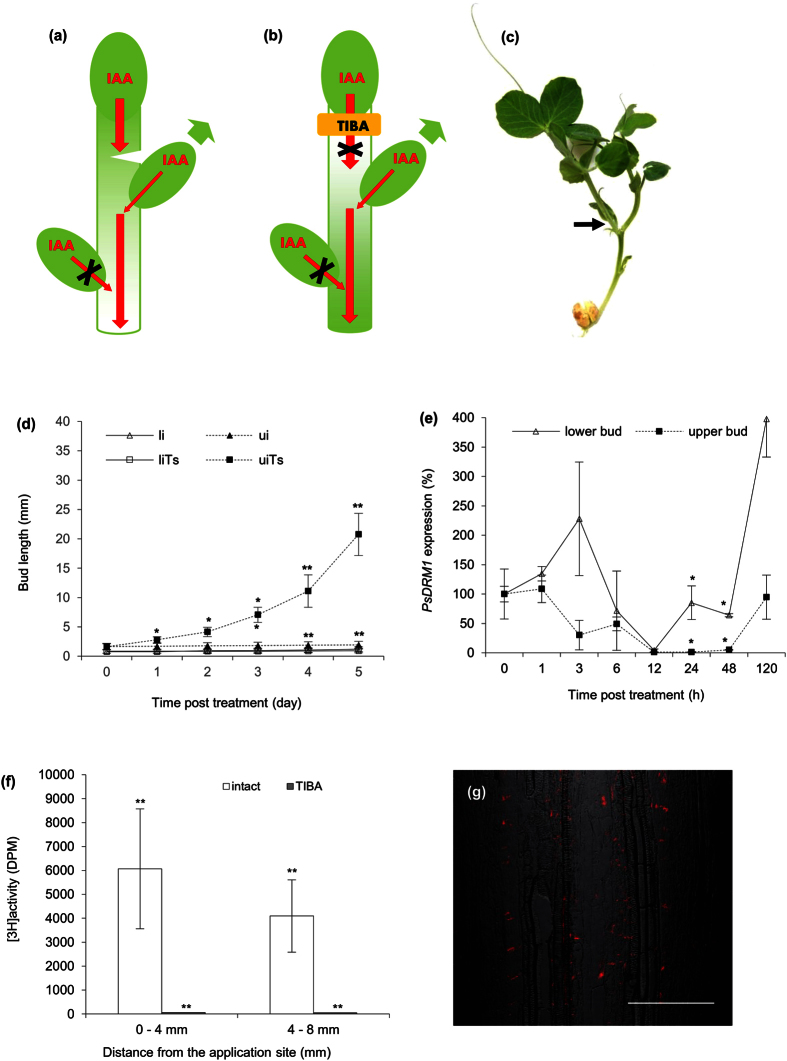
PAT Interruption in the primary stem releases buds from dormancy. **(a)** Scheme of plant wounded above the upper bud. Red arrows represent auxin (IAA) flow; red arrow crossed with black X represents disabled auxin flow. Green arrow represents bud outgrowth. Weakening stem auxin flow facilitated auxin export from the upper bud and its outgrowth. The lower bud remained arrested in dormancy by auxin loaded from the upper bud. **(b)** Scheme of intact plant subapically treated with TIBA-ring. Arrows as depicted in a). Auxin efflux inhibitor blocks auxin flow from the apex and the upper bud becomes a new auxin source, which continues to prevent auxin transport from the lower bud. **(c)** Wounded plant. Black arrow points to the lateral shoot formed from the upper axillary bud, above which the stem was incised. **(d)** Length of axillary buds and forming shoots, where: (li) lower bud of intact plants, (ui) same plants, upper bud, (liTs) lower bud of intact plants subapically treated with TIBA-ring, (uiTs) same treatment, upper bud. Statistically significant differences (identified by Student’s t-test) α = 0.05* and α = 0.01**. Error bars represent standard deviations (n = 60). **(e)** Relative expression of *PsDRM1* gene in the lower and upper axillary bud of intact plants subapically treated with TIBA-ring. Statistically significant differences (identified by Student’s t-test) α = 0.05* and α = 0.01**. Error bars represent standard deviations (n = 4). **(f)** [^3^H]-IAA transport from the apex in stem subapically treated with TIBA-ring was measured in two stem sections at a distance of 0–4 and 4–8 mm under the TIBA application site. Statistically significant differences (identified by Student’s t-test) α = 0.05* and α = 0.01**. Error bars represent standard deviations (n = 10). **(g)** PIN1 auxin efflux carrier immunoanalysis (red signal) in stem cells at TIBA-ring position exhibited no visible changes in organization. Stage 24 h after treatment. Scale bar, 100 μm.

The role of PAT system in stems for inhibition of axillary bud outgrowth was further tested by reducing intact plant auxin flow by imposing a deep stem wound. The incision positioned above the upper axillary bud (Fig. 3a) released this bud from dormancy while the lower bud remained arrested. The released upper bud subsequently formed a shoot and primary shoot also continued in growth (Fig. 3c). These axillary bud changes exhibited no relationship to altered nutrient supplies caused by wounding and cotyledon removal (Supplementary Fig. 1b,d).

As an alternative and supporting approach, we inhibited stem basipetal auxin flow by applying a ring of auxin efflux inhibitor 2,3,5-triiodobenzoic acid (TIBA) on the stem subapically, i.e., between the apex and upper axillary bud (Fig. 3b). The TIBA-ring effectively blocked stem auxin transport from the apex, as shown by radioactively labelled auxin ([^3^H]-IAA) application measurements (Fig. 3f). In addition, these results indicated that upper bud outgrowth was promoted, while lower buds remained arrested (Fig. 3d). *PsDRM1* dormancy marker expression confirmed the macroscopically observed bud dormancy status (Fig. 3e). Furthermore, PIN1 auxin efflux carrier immunodetection provided additional evidence that in intact plants subapically treated with a TIBA-ring (Fig. 3b) the polarized PIN1 carrier in procambial cell files established auxin export from the upper outgrowing buds (Supplementary Fig. 2a); however in arrested lower buds, signs of polarization were not observed (Supplementary Fig. 2b). In the stem itself, visible changes in PIN1 polarization on or adjacent to the TIBA-ring position were not detected (Fig. 3g). Same experimental setup with auxin efflux inhibitor 1-*N*-naphtylphtalamic acid (NPA) ring application led to identical results (Supplementary Fig. 3a,d,e) as with TIBA-ring, while protein synthesis inhibitor cycloheximide (CHX) ring did not promote bud outgrowth (Supplementary Fig. 4a,d) and did not block auxin transport from the apex (Supplementary Fig. 4e). Lengths of growing shoot apices measured from the subapically applied ring to the tip showed that TIBA and NPA content in the ring promoted shoot elongation above the application site in comparison to lanolin control and CHX-ring. Furthermore, the stem above TIBA- and NPA- ring was swollen (Supplementary Fig. 2c–g).

### Interruption of PAT between buds releases lower bud from dormancy

**Figure 4:**
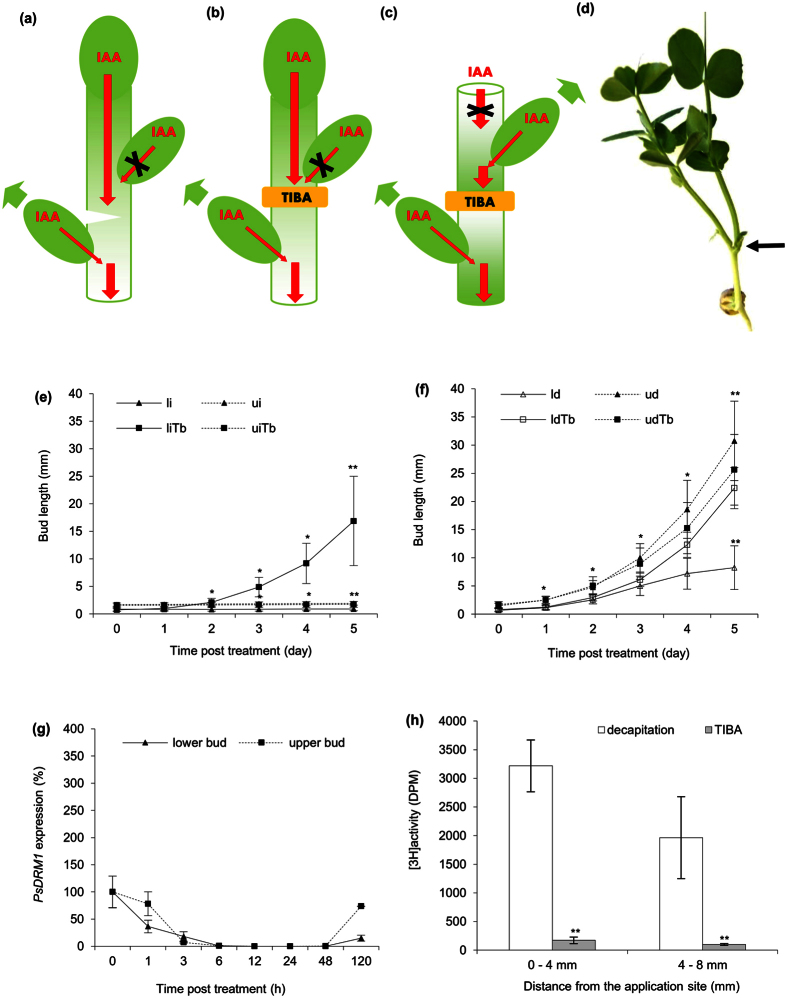
Interruption of PAT between buds releases lower bud from dormancy. **(a)** Scheme of plant wounded above the lower bud. Red arrows represent auxin (IAA) flow; red arrow crossed with black X represents disabled auxin flow. Green arrow represents bud outgrowth. Weakening stem auxin flow facilitated auxin export from the lower bud and its outgrowth. **(b)** Scheme of intact plant treated with TIBA-ring between the buds. Arrows as depicted in a). Auxin efflux inhibitor prevents auxin flow in the stem and allows auxin export from the lower bud and its outgrowth. **(c)** Scheme of decapitated plant treated with TIBA-ring between the buds. Arrows as depicted in a). Competition for outgrowth between buds is relinquished by TIBA, which isolates the lower bud from the auxin loaded by the upper bud, resulting in two equally growing shoots. **(d)** Wounded plant. Black arrow points to the lateral shoot was formed from the lower bud, above which the stem was incised. **(e)** Length of axillary buds and forming shoots, where: (li) lower bud of intact plants, (ui) same plants, upper bud, (liTb) lower bud of intact plants treated with TIBA-ring between the buds, (uiTb) same treatment, upper bud. Statistically significant differences (identified by Student’s t-test) α = 0.05* and α = 0.01**. Error bars represent standard deviations (n = 60). **(f)** Length of axillary buds and forming shoots, where: (ld) lower bud of decapitated plants, (ud) same plants, upper bud, (ldTb) lower bud of decapitated plants treated with TIBA-ring between the buds, (udTb) same treatment, upper bud. Statistically significant differences (identified by Student’s t-test) α = 0.05* and α = 0.01**. Error bars represent standard deviations (n = 60). **(g)** Relative expression of *PsDRM1* gene in lower and upper buds of decapitated plants treated with TIBA-ring between the buds. Statistically significant differences (identified by Student’s t-test) α = 0.05* and α = 0.01**. Error bars represent standard deviations (n = 4). **(h)** [^3^H]-IAA transport in decapitated stems from shoots formed from upper axillary buds measured in two stem sections at distance of 0–4 and 4–8 mm under the TIBA-ring between the buds. Statistically significant differences (identified by Student’s t-test) α = 0.05* and α = 0.01**. Error bars represent standard deviations (n = 10).

Auxin flow was disrupted in the primary stem between axillary buds in intact plants to examine bud initiation. Deep stem wound positioned above the lower axillary bud (Fig. 4a) (or wherever between the upper and lower bud) released the lower bud from inhibition that subsequently formed a shoot, while the upper bud remained inhibited. In addition to the lower bud outgrowth, the primary shoot also continued its growth (Fig. 4d). This outgrowth pattern was not affected by changed nutrient supplies caused by wounding and cotyledon removal (Supplementary Fig. 1c,e).

Instead of the incision, a TIBA-ring was applied on the stem between the buds of intact plant (gjj). Results were consistent with those previously observed, where the lower bud exhibited outgrowth, while the upper remained arrested (Fig. 4e). Auxin transport was further examined by applying a TIBA-ring between the axillary buds of decapitated plants to inhibit stem auxin transport (Fig. 4c). TIBA as an auxin efflux inhibitor isolated the upper and lower axillary buds; monitored and confirmed by the blocked stem [^3^H]-IAA transport below the TIBA-ring (Fig. 4h), however, [^3^H]-IAA export from the forming lower shoots was not affected while from the upper shoots analysed above the TIBA-ring was reduced (Supplementary Fig. 5a,c). Further, the similar dynamics of *PsDRM1* expression in both axillary buds confirmed their independent outgrowth (Fig. 4g). TIBA-ring application resulted in two equally growing shoots, without any signs of sub- or super-ordination (Fig. 4f). NPA-ring application led to same results (Supplementary Fig. 3b,c,f,g,h) as TIBA-ring, while CHX-ring on intact plants neither promote bud outgrowth (Supplementary Fig. 4b) nor interfere with bud outgrowth pattern after decapitation (Supplementary Fig. 4c), however, reduced the stem auxin flow from the upper forming shoot (Supplementary Fig. 4g).

### Inhibition of PAT from the upper bud releases lower bud from dormancy

**Figure 5:**
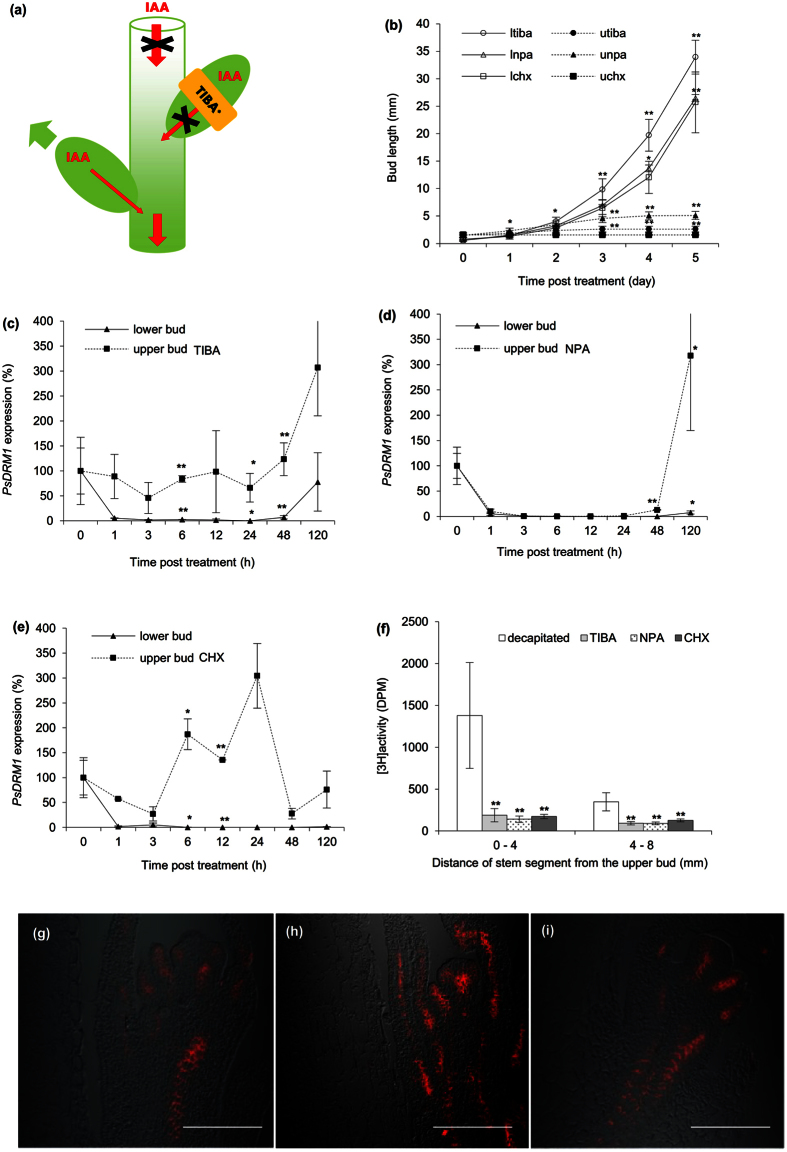
Inhibition of PAT from the upper bud releases lower bud from dormancy. **(a)** Scheme of decapitated plant treated with TIBA-, NPA-, or CHX-ring on the upper bud (asterisk indicates NPA or CHX was also applied). Red arrows represent auxin (IAA) flow; red arrows crossed with black X represent disabled auxin flow. Green arrow represents bud outgrowth. Treated bud outgrowth was inhibited by a mechanism associated with each applied chemical, which resulted in the lower bud becoming a new auxin source and developing a shoot. **(b)** Length of axillary buds and forming shoots, where: (ltiba) lower bud of decapitated plants, when upper bud was treated with TIBA, (utiba) same treatment, upper bud, (lnpa) lower bud of decapitated plants, when upper bud was treated with NPA, (unpa) same treatment, upper bud, (lchx) lower bud of decapitated plants, when upper bud was treated with CHX, (uchx) same treatment, upper bud. Statistically significant differences (identified by Student’s t-test) α = 0.05* and α = 0.01**. Error bars represent standard deviations (n = 60). **(c–e)** Relative expression of *PsDRM1* gene in lower and upper axillary buds following TIBA **(c)**, NPA **(d),** and CHX **(e)** treatments in upper buds of decapitated plants. Statistically significant differences (identified by Student’s t-test) α = 0.05* and α = 0.01**. Error bars represent standard deviations (n = 4). **(f)** [^3^H]-IAA export from upper axillary buds, measured in two stem sections, from 0–4 and 4–8 mm under the upper bud. Decapitation facilitated auxin export, while TIBA, NPA and CHX reduced auxin flow. Statistically significant differences (identified by Student’s t-test) α = 0.05* and α = 0.01**. Error bars represent standard deviations (n = 10). **(g–i)** PIN1 auxin efflux carrier immunoanalysis (red signal) in axillary buds showed TIBA **(g)**, NPA **(h)**, and CHX **(i)** lanolin paste treatments did not prevent polarization following decapitation. Stages 24 h after treatment. Scale bar, 100 μm.

Auxin export inhibition specifically from the upper bud was used to intervene with bud competition. TIBA-ring effects applied on the upper buds of decapitated plants were tested (Fig. 5a). As expected, continuously growing shoots were formed from lower buds, while the treated upper buds returned to the dormant state following an initial growth period (Fig. 5b). If instead the TIBA-ring NPA-ring was applied (Fig. 5a), growth of lower bud and inhibition of upper bud (Fig. 5b) was similar to TIBA treated plants. Application of the protein synthesis inhibitor CHX-ring (Fig. 5a) had again same effect, and moreover, the treated upper buds were arrested completely (Fig. 5b). Decreased *PsDRM1* expression reflected growth status of lower buds and the several fold-increased expression in the treated upper buds corresponded with their return to dormancy (Fig. 5c–e). [^3^H]-IAA export assays from the treated upper buds of decapitated plants showed that TIBA, NPA, and CHX were strong inhibitors of [^3^H]-IAA export (Fig. 5f). In addition, TIBA-, NPA-, or CHX-ring applied on decapitated stem above the upper bud did not affect the [^3^H]-IAA export from the upper bud (Supplementary Fig. 5b,d). Application of TIBA and NPA to axillary buds did not prevent initial PIN1 polarization or visibly affect PIN1 polar localization (Fig. 5g,h). In addition, immunoanalysis of CHX-treated buds, which were effectively arrested, showed normal PIN1 polarization (Fig. 5i).

## Discussion

In plants with strong apical dominance, the shoot apex supplies the primary stem with auxin, and inhibits outgrowth of axillary buds. Saturated polar auxin flow in the primary stem does not become a sink for auxin flux from axillary buds, therefore PIN auxin efflux carriers in buds remained unpolarized. Removal of the shoot apex initiates axillary bud growth. This release from dormancy and outgrowth was accompanied by PIN carrier polarization, enabling auxin export from the buds. Previously we showed that inhibition of bud outgrowth was caused by competition between apical and lateral auxin sources (shoot apex versus axillary bud) for primary auxin transport channels converging in the stem auxin stream^10^. Subsequently, we subjected the axillary buds in our two-nodal-bud model system to a much closer examination and observed competition between buds. This result suggested that the competitive nature for dominance also applies to axillary buds after decapitation, i.e., upper buds inhibited lower buds, but dominance was not imposed immediately following decapitation. Thus, morphology and the *PsDRM1* marker^13^ demonstrated competition for dominance between axillary buds when released from dormancy, i.e., the upper bud gained dominance over the lower bud during time post treatment. The importance of basipetal auxin flow in stems for bud outgrowth regulation was evaluated using de-etiolated plants with long internodes. *BRC1* (*BRANCHED1*) is a putative integrator of different branching pathways in Arabidopsis and its expression changes rapidly following decapitation^14^. We observed that the same expression dynamics applied in our pea model. Given average polar auxin transport (PAT) velocity is ~10 mm h^−1 ^15, this result suggested auxin depletion or at least its decrease from the missing auxin source at the stem apex had an impact on the timing of bud outgrowth. Similar effects of the stem on bud growth were reported^16^ in soybean. Buds located on the basal ends of excised stem segments showed partly inhibited growth compared with buds on the apical ends of segments. However, several more recent studies performed on 20-day-old pea plants under different experimental regimes reported that onset of axillary bud outgrowth following decapitation or girdling was not always accompanied by local auxin depletion in the stem^17^,^18^,^19^,^20^,^21^. Nonetheless, our observations in the two-nodal-bud pea system showed a high correlation between the axillary buds’ release from dormancy and known stem auxin depletion resulting from decapitation.

There are indications that NPA have a rapid restricting systemic effect on basipetal auxin flow^21^. In the used decapitated two-nodal-bud pea model system nor auxin efflux inhibitors NPA or TIBA nor protein synthesis inhibitor CHX applied on stem showed effect on auxin export from the shoots formed below the application site. However, in the shoots formed above application site the amount of transported [^3^H]-IAA was reduced by approx. 50%, but this reduced PAT flow was still enough for unaffected long-term growth. The observed promoted internode elongation and swelling if TIBA or NPA was applied subapically to intact plants are typical results of high auxin content^22^,^23^ giving indirect evidence that despite the reduced PAT from the apex^21^ the amount of flowing auxin was still enough to produce the physiological effects.

Interruption of polar auxin flow in the primary stem by auxin efflux inhibitor TIBA^24^ or NPA^25^,^26^ or by lateral incision released axillary buds from dormancy in the presence of the shoot apex. The outgrowing axillary bud (lower or upper) was consistently the bud above which the primary stem auxin flux was interrupted. This outgrowth pattern was not influenced if nutrient supplies were changed by cotyledon removal. Furthermore, considering the roots as main source of strigolactone (SL)^27^,^28^,^29^, demonstrated as effective inhibitor of bud outgrowth^21^, it can be hypothesized that incision above the bud could direct more acropetally moving SL into this bud and cause its inhibition. Nonetheless, this bud was released from inhibition and formed a shoot. This finding supports the hypothesis that SL inhibits bud growth only in the presence of auxin in the main stem^9^. In addition, the acropetal flow of cytokinins in the stem^30^ restricted by incision could also enter the bud and promote bud outgrowth initiation. These results indicated sustainable auxin flow in the primary stem from the apex was required to maintain axillary bud dormancy and auxin flow interruption or inhibition released the upper bud from dormancy, as demonstrated by bud outgrowth, dormancy markers, and PIN1 auxin carrier polarization. These results are also congruent with the hypothesis that auxin flow from more apical plant parts controls the dormancy status of axillary buds. Furthermore, the typical result of competition for dominance was initiated by decapitation, where the upper axillary bud outcompeted the lower bud. This competition pattern was not altered by cotyledon removal. Competition ceased by interruption of auxin flow between upper and lower competing axillary buds, resulting in two long-term equally growing shoots. Similarly, the position-predetermined “winning” upper bud can be disqualified from the competition game by inhibition of the auxin flow from it, by auxin efflux inhibitors or protein synthesis inhibitor. TIBA blocks PIN trafficking between the plasma membrane and endosomal compartments more generally, leaving PIN1 accumulation at the plasma membrane unaffected^24^. However, PIN proteins or PIN cycling are apparently not directly affected by NPA^31^,^32^. Despite these described effects of TIBA on PIN trafficking, TIBA or NPA applied to axillary buds did not affect initial PIN1 polarization. This result was unexpected, but effective chemical concentrations in plant tissues might be sufficient to inhibit auxin export and initial bud outgrowth, but not affect steady-state PIN localization. The protein synthesis inhibitor CHX also interferes with auxin transport^33^,^34^. However, the CHX-treated effectively arrested buds showed normal initial PIN1 polarization. Similar results were reported in Arabidopsis roots, where CHX exhibited no detectable effects on PIN1 at the plasma membrane^35^. These experiments demonstrated that chemical inhibition of auxin export from buds does not necessarily impact PIN1 polarization in treated buds. These results suggested not only PIN polarization, but more importantly the capacity of auxin export from buds is required for sustained outgrowth of axillary shoots.

Based on the auxin canalization theory, establishment of an effective auxin transport channel following interruption of stem auxin flow leads to redirection of the auxin source-sink pattern from the original primary-apex-root pathway to a new secondary-apex-root pathway. Developmental processes involved in the new vascular strands are consistent with the directed auxin flow. The auxin flow source is acting as a sink for assimilates and nutrients essential for development and further reproduction in the outgrowing shoot’s body mass. More detailed studies on the influence of other hormones and exogenous factors on auxin flow-mediated bud competition will likely elucidate its mechanisms.

## Methods

### Plant material, growth conditions, inhibitors and hormonal treatment

Pea plants (*Pisum sativum* L.) cv. Vladan were grown in perlite soaked with Richter’s nutrient solution in a growth chamber at 20 °C/18 °C day/night temperatures, under a 16 h day/8 h night cycle photoperiod and light intensity 150 μmol m^−2^ s^−1^. Age of plants was 7 days after sowing (DAS), experimental variants were intact, decapitated 10 mm above the upper bud, or identically decapitated and decotyledoned. Following protocols: i) axillary bud and apical shoot length measurement; ii) axillary bud gene expression analyses; iii) PIN1 protein immunolocalization assays; iv) polar auxin transport capacity assays were used. Furthermore, 7 DAS plants or 7 DAS decotyledoned plants were administered with a deep lateral incision above the upper axillary bud or between the lower and upper axillary buds and used for axillary bud length measurements (henceforth wounded plants). Ten DAS de-etiolated plants decapitated 90 mm (long stump) or 5 mm (short stump) above the upper axillary bud were used for gene expression analyses in the upper axillary buds.

Water lanolin paste (control) or paste containing 1% 2,3,5-triiodobenzoic acid (TIBA), 1% 1-*N*-naphtylphtalamic acid (NPA), or 1% cycloheximide (CHX) was applied as a ring on the stem, subapically 5 mm below apex, 5 mm above the upper bud or between the upper and lower axillary bud of intact and decapitated plants. Water lanolin paste (control) or paste containing 1% TIBA, 1% NPA, or 1% CHX were applied on the upper axillary bud 4 h before decapitation. The upper and lower axillary buds were used to explore gene expression and PIN1 protein localization. For bud length measurements, 60 plants in two biological replicates were used for each treatment.

### Gene expression analysis (RNA extraction, cDNA synthesis, and quantitative Real-Time PCR)

Bud samples were harvested and ground in liquid nitrogen. Total RNA for each sample was isolated from 30 buds using the RNeasy Plant Mini Kit (Qiagen) following the manufacturer’s protocol. A DNase treatment with the RNase-free DNase Set (Qiagen) was carried out for 15 min at 25 °C. Total cDNA was synthesized from 0.5 μg of total RNA using the Superscript III cDNA kit (Invitrogen).

Real-Time PCR (qPCR) was performed using LC 480 SYBR Green I Master Mix (Roche Diagnostics) with the following gene specific primers: *PsDRM1*: *PsDRM1* forward (5′-AAC TCA CCA CCA CCC TCA AAG ATG-3′) and *PsDRM1* reverse (5′-GAT GTA GAC ACG TGG CAG AAG ATG-3′); *PsBRC1: PsBRC1* forward (5′-AGG CAA GAG AAA GAG CAA GG-3′) and *PsBRC1* reverse (5′-TTG CAT TGC TTT GAG TTT GA-3′). Cycling conditions for amplification were 95 °C for 10 min, 40 cycles of 95 °C for 15 s, 58 °C for 15 s (for *Psβ-tubulin, PsActin, PsEF1-α* and *PsDRM1*) or 62 °C for 15 s (for *PsBRC1*), and 72 °C for 15 s. A gene expression normalization factor was calculated (Microsoft Excel geNorm, 2002) based on *Psβ-tubulin, PsActin*, and *PsEF1-α* (primer sequences^36^) expression levels. Two biological replicates were analyzed in duplicates. The mean value and standard deviations were determined from replications of each variant. A Student’s t-test was performed to test for significant differences between individual variants.

### Immunolocalization of PIN1 protein

Immunolocalization was performed on longitudinal stem segments or stem segments containing the lower or upper axillary buds collected 24 h after treatment, with 10 replicate segments from each sample type, following the published protocol^37^. The anti-Arabidopsis-PIN1 antibody also recognizes the homologous PIN protein in pea, which is presumed to be a PIN1 functional ortholog based on expression similarity and localization signal to Arabidopsis^38^. The following antibodies and dilutions were used: anti-PIN1 (1:500) and CY3-conjugated anti-rabbit secondary antibody (1:500). Samples were viewed under a confocal laser scanning microscope Fluoview 200 (Olympus) using UPlanFI 20x/0.5 objective at room temperature. Images were acquired using Fluoview 5.0 software.

### Polar auxin transport capacity assay

Plants were treated with 0.5 or 1 μl of [5-^3^H]-indole-3-acetic acid (American Radiolabeled Chemicals, 925 Gbq mmol^−1^, 6666 Bq μl^−1^) diluted in a 50% ethanol solution for auxin export assays. For each experimental variant stem segment samples were collected from 10 plants. In all stem segment variants samples were incubated in a dioxane-based liquid scintillator cocktail overnight. The [^3^H] activity in stem segments was measured with a scintillation spectrophotometer Packard TRI/Carb 2000 (Packard).

Auxin export was assessed from the apex of plants with a subapical ring application around the stem of pure lanolin (control) or 1% TIBA, 1% NPA or 1% CHX lanolin paste. Twenty-four h after treatment, 1 μl of [^3^H]-IAA was applied to the apex tip and 2 h later, the stem under the application site was cut into 4 mm segments of 0–4 and 4–8 mm.

Auxin export from outgrowing shoots was examined on plants treated with pure lanolin (control) or 1% TIBA, 1% NPA or 1% CHX lanolin paste between upper and lower axillary buds as a ring around the stem, and immediately decapitated 10 mm above the upper axillary bud. Three days later, 1 μl of [^3^H]-IAA was applied to the tip of the upper outgrowing shoot. After 2 h, the stem under the application site was cut into 4 mm segments of 0–4 and 4–8 mm or into 6 mm segments below the forming upper and lower shoot.

Auxin export was evaluated from upper axillary buds by application of lanolin paste (control) or 1% TIBA, 1% NPA, 1% CHX lanolin paste or control lanolin past ring at the base of the upper axillary bud. Plants were decapitated 10 mm above the upper bud 4 h following treatment. Six hours after decapitation, 0.5 μl of [^3^H]-IAA was applied to the tip of the upper axillary bud; and following 1.5 h, the stem under the upper axillary bud was cut into 4 mm segments of 0–4 and 4–8 mm. Furthermore, auxin export from upper buds was examined on plants decapitated 10 mm above the upper bud and treated with pure lanolin (control) or 1% TIBA, 1% NPA or 1% CHX lanolin paste 5 mm above the upper axillary bud. 24 h after this treatment 0.5 μl of [^3^H]-IAA was applied to the tip of the upper bud; and following 1.5 h, the stem under the upper axillary bud was cut into 4 mm segments of 0–4 and 4–8 mm.

## Additional Information

**How to cite this article**: Balla, J. *et al*. Auxin flow-mediated competition between axillary buds to restore apical dominance. *Sci. Rep.* **6**, 35955; doi: 10.1038/srep35955 (2016).

**Publisher’s note:** Springer Nature remains neutral with regard to jurisdictional claims in published maps and institutional affiliations.

## Supplementary Material

Supplementary Information
