# Supplementary material for: Auxin flow-mediated competition between axillary buds to restore apical dominance
Source: Sci Rep. 2016 Nov 8;6:35955. doi: 10.1038/srep35955 (PMC5099894; doi:10.1038/srep35955)
Supplement: Supplementary Information [file srep35955-s1.pdf]

Supplementary Information

Auxin flow-mediated competition between axillary buds to restore apical dominance

Jozef Balla[1,2+*], Zuzana Medveďová[1,4+], Petr Kalousek[2], Natálie Matiješčuková[1], Jiří Friml[3], Vilém Reinöhl[1] and Stanislav Procházka[1]

[+]These authors contributed equally to this work.

*Correspondence and requests for materials should be addressed to J.B. (email: xballa@mendelu.cz)

Supplementary Figure S1.

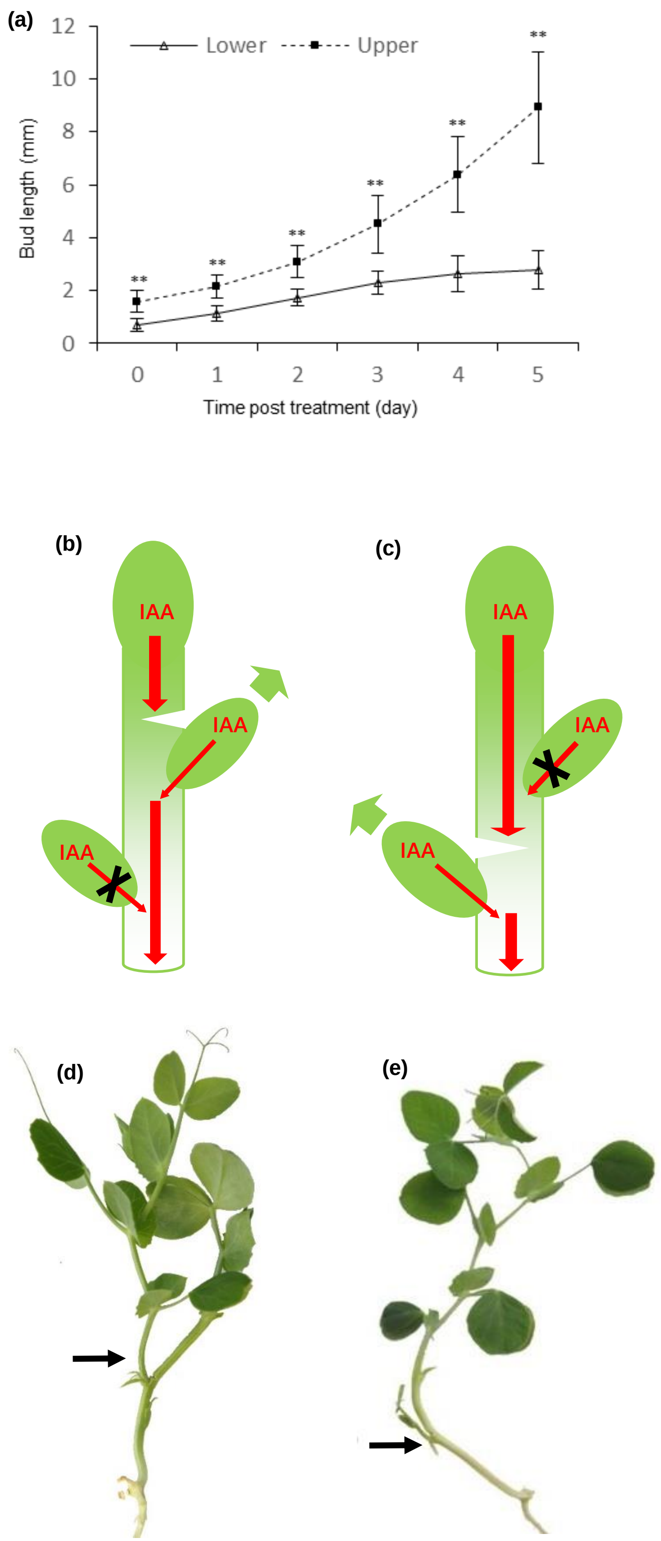

Supplementary Figure S1. Interruption of PAT releases buds on decotyledoned plants.

(a) Length of upper and lower axillary buds and forming shoots on decapitated and decotyledoned plants. Statistically significant differences (identified by Student's t-test): $\alpha=0.05$* and $\alpha=0.01$**. Error bars represent standard deviations (n=60).

(b) Scheme of wounded decotyledoned plant with lateral incision above the upper bud. Red arrows represent auxin (IAA) flow; red arrow crossed with black X represents disabled auxin flow. Green arrow represents bud outgrowth. Weakening stem auxin flow allowed auxin export from the upper bud and its outgrowth. The lower bud remained arrested in dormancy by auxin loading from the upper bud.

(c) Scheme of wounded decotyledoned plant with lateral incision above the lower bud. Arrows as depicted in a). Weakening stem auxin flow facilitated auxin export from the lower bud and its outgrowth. The upper bud remained further arrested by auxin loading from the apex.

(d) Wounded decotyledoned plant. Black arrow points to the lateral shoot formed from the upper axillary bud, above which the stem was incised.

(e) Wounded decotyledoned plant. Black arrow points to the lateral shoot formed from the lower axillary bud, above which the stem was incised

Supplementary Figure S2.

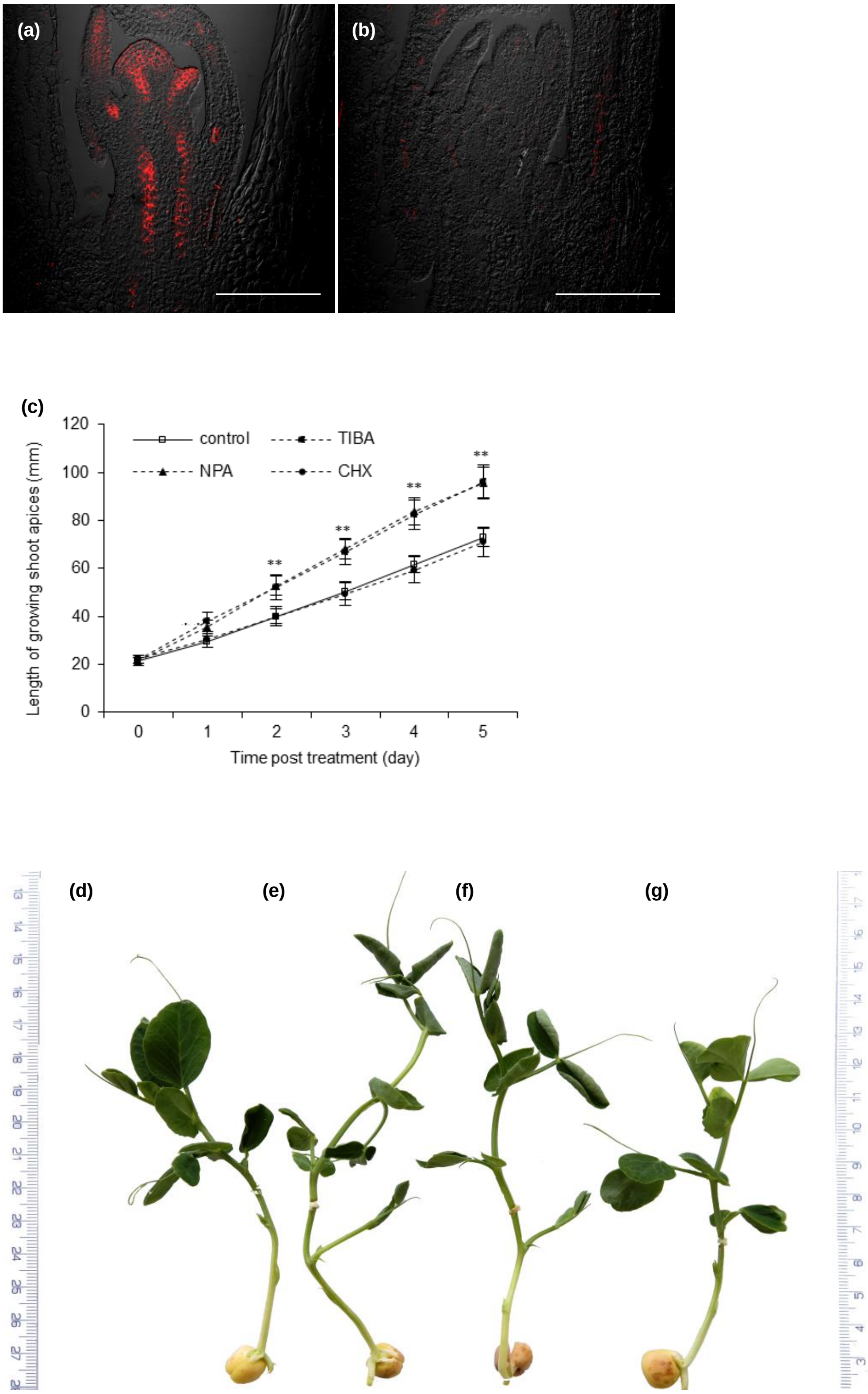
(a)
(b)
(c)
control
TIBA
NPA
CHX
Length of growing shoot apices (mm)
120
100
80
60
40
20
0
**
0
1
2
3
4
5
Time post treatment (day)
(d)
(e)
(f)
(g)

Supplementary Figure S2. Subapical TIBA, NPA, CHX and control lanolin treatment on primary stem has different effect for bud outgrowth.

(a and b) Immunoanalysis of PIN1 auxin efflux carriers (red signal) showed polar localization in procambial cells of outgrowing axillary buds **(a)**, lack of localization in procambial cells of inhibited axillary buds **(b)**. Scale bar, 100 μm.

(c) Lengths of growing shoot apices measured from the subapically applied ring of TIBA, NPA, CHX and control lanolin to the tip. Statistically significant differences (identified by Student's t-test) $\alpha=0.05$* and $\alpha=0.01$**. Error bars represent standard deviations (n=60).

(d and g) Plants subapically treated with control lanolin ring **(d)**, or lanoline ring containing TIBA **(e)**, NPA **(f)** or CHX **(g)**.

Supplementary Figure S3.

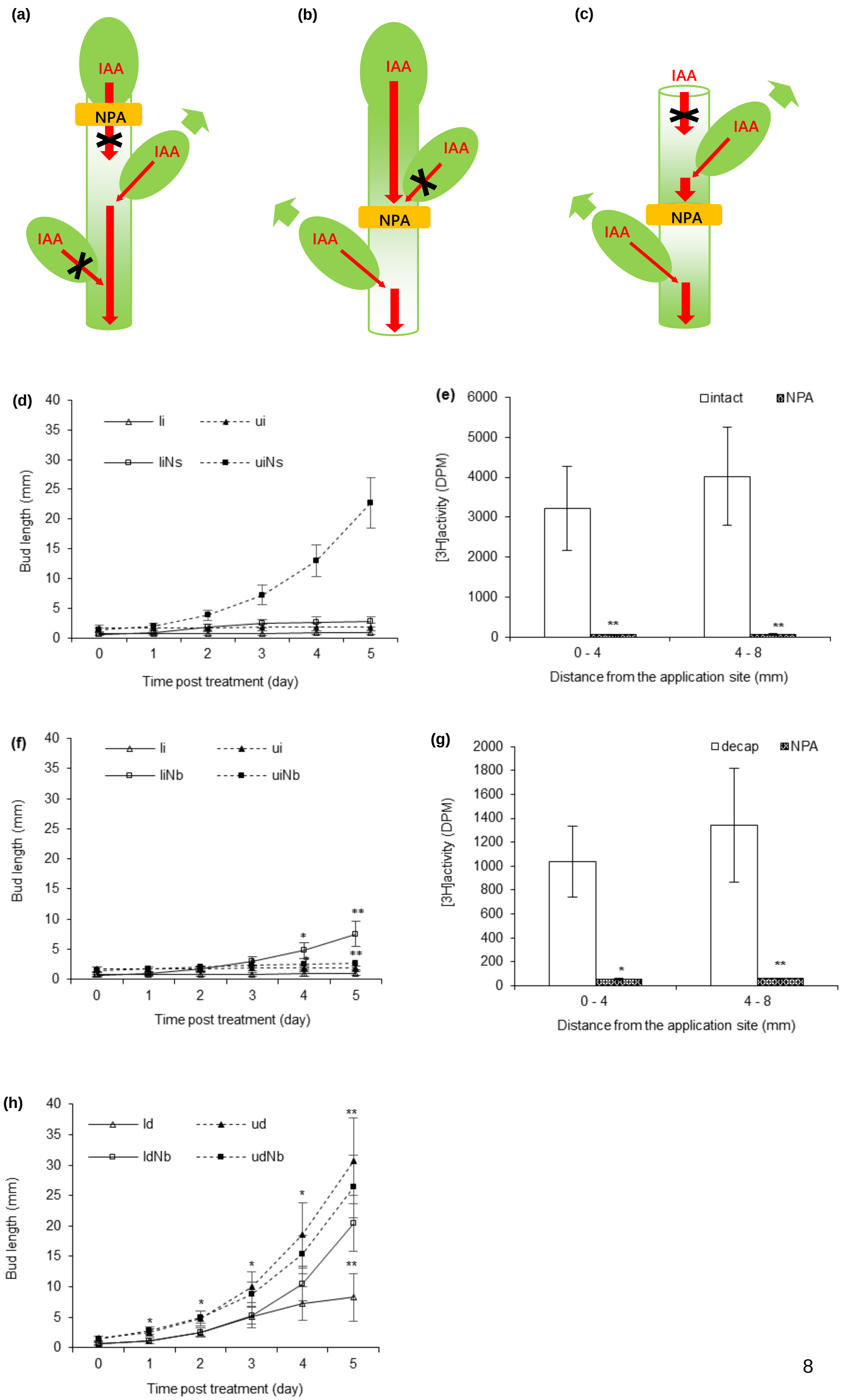

(a)
IAA
NPA
IAA
IAA
(b)
IAA
IAA
NPA
IAA
(c)
IAA
IAA
NPA
IAA
(d)
li
ui
liNs
uiNs
Bud length (mm)
Time post treatment (day)
(e)
intact
NPA
[3H]activity (DPM)
0 - 4
4 - 8
Distance from the application site (mm)
(f)
li
ui
liNb
uiNb
Bud length (mm)
Time post treatment (day)
(g)
decap
NPA
[3H]activity (DPM)
0 - 4
4 - 8
Distance from the application site (mm)
(h)
ld
ud
ldNb
udNb
Bud length (mm)
Time post treatment (day)

Supplementary Figure S3. NPA treatment on primary stem interrupts PAT.

(a) Scheme of plant subapically treated with NPA-ring. Red arrows represent auxin (IAA) flow; crossed red arrows represent disabled auxin flow. Green arrow represents bud outgrowth. Weakening stem auxin flow facilitated auxin export from the upper bud and its outgrowth. Lower bud remained arrested in dormancy by auxin loaded from the upper bud.

(b) Scheme of intact plant treated with NPA-ring. Arrows as in a). NPA prevents auxin flow in the stem and allows auxin export from the lower bud.

(c) Scheme of decapitated plant treated with NPA-ring. Arrows as in a). NPA isolates the lower bud from auxin loaded by the upper bud, resulting in two equally growing shoots.

(d) Length of axillary buds and forming shoots, where: **(li)** lower bud of intact plants, **(ui)** same plants, upper bud, **(liNs)** lower bud of intact plants subapically treated with NPA-ring, **(uiNs)** same treatment, upper bud. Statistically significant differences (Student's t-test) $\alpha=0.05$* and $\alpha=0.01$**. Error bars represent standard deviations (n=60).

(e) [^{3}H]-IAA transport from the apex in stem subapically treated with NPA-ring measured in two stem sections at distance 0-4 and 4-8 mm under NPA-ring. Statistically significant differences (Student's t-test) $\alpha=0.05$* and $\alpha=0.01$**. Error bars represent standard deviations (n=10).

(f) Length of axillary buds and forming shoots, where: **(li)** lower bud of intact plants, **(ui)** same plants, upper bud**, (liNb)** lower bud of intact plants treated with NPA-ring between buds, **(uiNb)** same treatment, upper bud. Statistically

significant differences (Student’s t-test) α=0.05* and α=0.01**. Error bars represent standard deviations (n=60).

(g) [^{3}H]-IAA transport in decapitated stems from upper axillary bud-formed shoots measured in two stem sections at distance 0-4 and 4-8 mm under NPA-ring between buds. Statistically significant differences (identified by Student’s t-test) α=0.05* and α=0.01**. Error bars represent standard deviations (n=10).

(h) Length of axillary buds and forming shoots, where: **(ld)** lower bud of decapitated plants, **(ud)** same plants, upper bud, **(ldNb)** lower bud of decapitated plants treated with NPA-ring between buds, **(udNb)** same treatment, upper bud. Statistically significant differences (Student’s t-test) α=0.05* and α=0.01**. Error bars represent standard deviations (n=60).

Supplementary Figure S4.

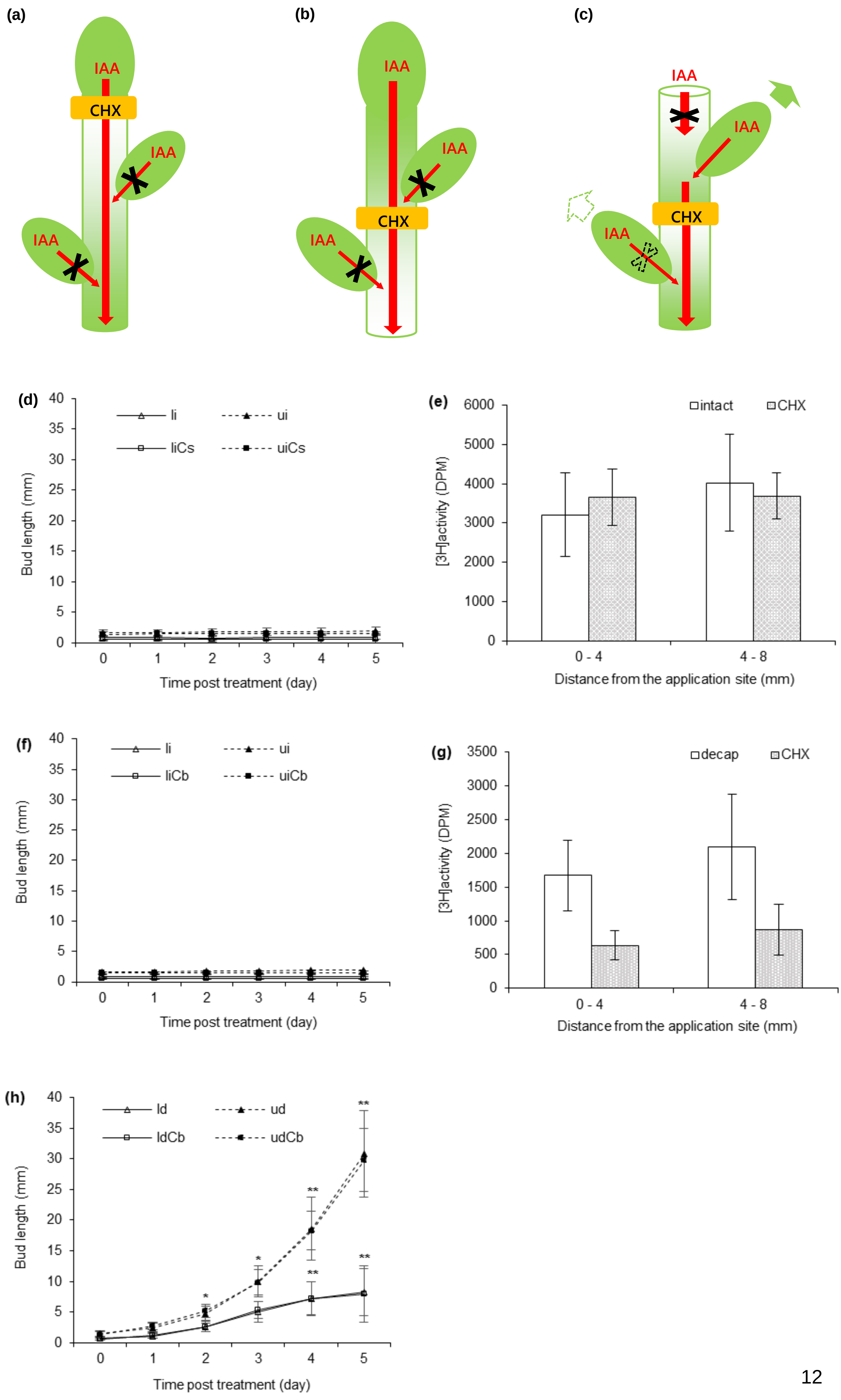
(a)
IAA
CHX
IAA
IAA
(b)
IAA
IAA
CHX
IAA
(c)
IAA
IAA
CHX
IAA
(d)
li
ui
liCs
uiCs
Bud length (mm)
Time post treatment (day)
(e)
intact
CHX
[3H]activity (DPM)
0 - 4
4 - 8
Distance from the application site (mm)
(f)
li
ui
liCb
uiCb
Bud length (mm)
Time post treatment (day)
(g)
decap
CHX
[3H]activity (DPM)
0 - 4
4 - 8
Distance from the application site (mm)
(h)
ld
ud
ldCb
udCb
Bud length (mm)
Time post treatment (day)

Supplementary Figure S4. CHX treatment on primary stem does not reduce PAT significantly.

(a) Scheme of plant subapically treated with CHX-ring. Red arrows represent auxin (IAA) flow; crossed red arrows represent disabled auxin flow. CHX-ring does not reduce auxin flow from apex. This flow prevents auxin canalization and its export, keeping the buds in dormancy.

(b) Scheme of intact plant treated with CHX-ring. Arrows as in a). CHX-ring does not reduce stem auxin flow and axillary buds remain arrested in dormancy.

(c) Scheme of decapitated plant treated with CHX-ring. Red arrows as in a). Dashed crossed red arrow represents temporary auxin flow. Green arrow represents bud outgrowth, dashed green arrow represents temporary outgrowth. CHX does not interfere with bud competition.

(d) Length of axillary buds and forming shoots, where: **(li)** lower bud of intact plants, **(ui)** same plants, upper bud, **(liCs)** lower bud of intact plants subapically treated with CHX-ring, **(uiCs)** same treatment, upper bud. Statistically significant differences (Student’s t-test) α=0.05* and α=0.01**. Error bars represent standard deviations (n=60).

(e) [^{3}H]-IAA transport from the apex in stem subapically treated with CHX-ring measured in two stem sections at distance 0-4 and 4-8 mm under CHX-ring. Statistically significant differences (Student’s t-test) α=0.05* and α=0.01**. Error bars represent standard deviations (n=10).

(f) Length of axillary buds and forming shoots, where: **(li)** lower bud of intact plants, **(ui)** same plants, upper bud, **(liCb)** lower bud of intact plants treated with CHX-ring between buds, **(uiCb)** same treatment, upper bud. Statistically

significant differences (Student's t-test) α=0.05* and α=0.01**. Error bars represent standard deviations (n=60).

(g) [^{3}H]-IAA transport in decapitated stems from upper axillary bud-formed shoots measured in two stem sections at distance 0-4 and 4-8 mm under CHX-ring between buds. Statistically significant differences (Student's t-test) α=0.05* and α=0.01**. Error bars represent standard deviations (n=10).

(h) Length of axillary buds and forming shoots, where: **(ld)** lower bud of decapitated plants, **(ud)** same plants, upper bud, **(ldCb)** lower bud of decapitated plants treated with CHX-ring between buds, **(udCb)** same treatment, upper bud. Statistically significant differences (Student's t-test) α = 0.05* and α=0.01**. Error bars represent standard deviations (n=60).

Supplementary Figure S5.

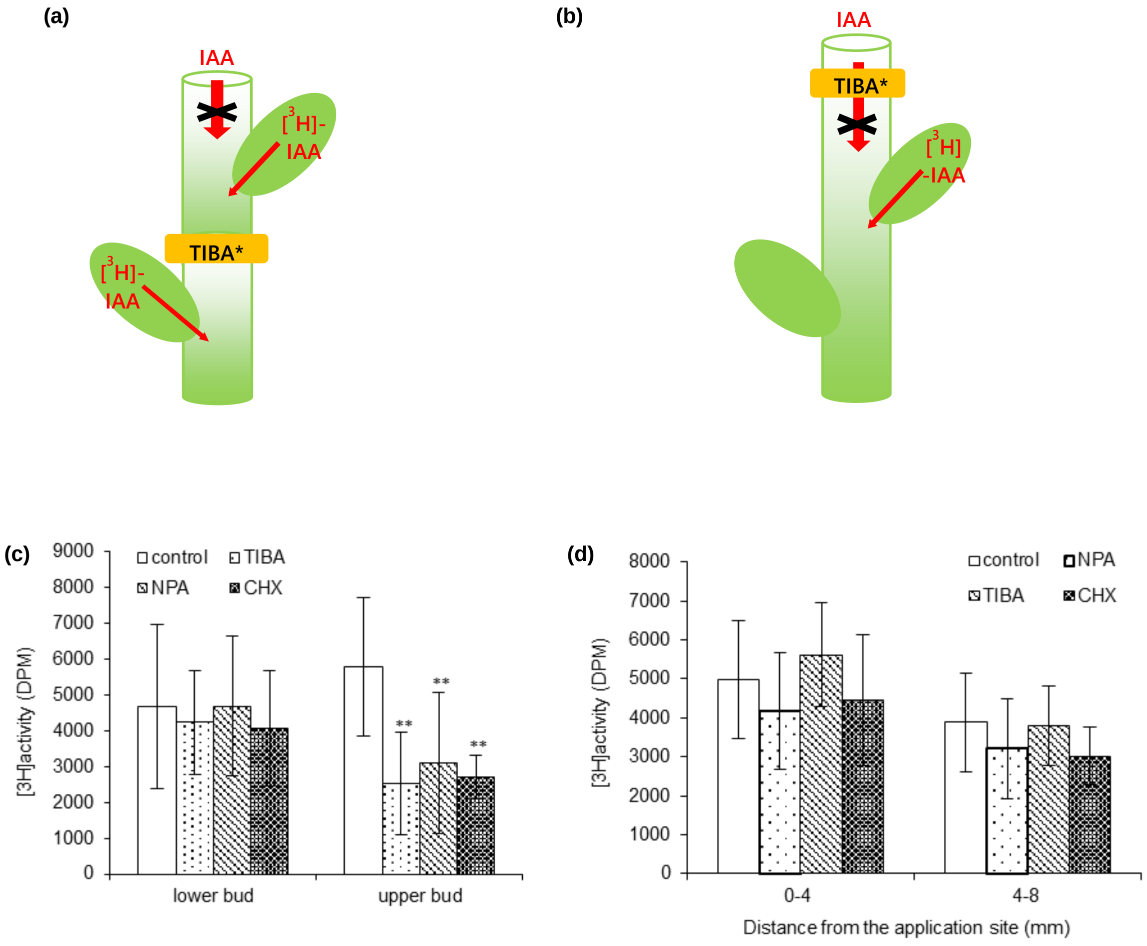
(a)
IAA
[3H]-IAA
TIBA*
[3H]-IAA
(b)
IAA
TIBA*
[3H]-IAA
(c)
control
TIBA
NPA
CHX
[3H]activity (DPM)
9000
8000
7000
6000
5000
4000
3000
2000
1000
0
**
**
**
lower bud
upper bud
(d)
control
NPA
TIBA
CHX
[3H]activity (DPM)
8000
7000
6000
5000
4000
3000
2000
1000
0
0-4
4-8
Distance from the application site (mm)

Supplementary Figure S5. Effect of TIBA, NPA, CHX and lanolin control treatment on [^{3}H]-IAA transport in decapitated primary stem.

(a) Scheme of decapitated plant treated with TIBA-ring between buds. Asterisk represents that effect of NPA, CHX and lanolin control in same experimental setup was also tested. Red arrows represent [^{3}H]-IAA flow; red arrow crossed with black X represents disabled auxin (IAA) flow.

(b) Scheme of decapitated plant treated with TIBA-ring above upper bud. Asterisk represents that effect of NPA, CHX and lanolin control in same experimental setup was also tested. Arrows as depicted in a).

(c) [^{3}H]-IAA transport in decapitated and TIBA, NPA, CHX and lanolin control treated (between upper and lower bud) stems from shoots formed from upper and lower axillary buds measured in 6 mm stem sections directly below the shoots. Statistically significant differences (identified by Student's t-test) $\alpha=0.05$* and $\alpha=0.01$**. Error bars represent standard deviations (n=10).

(d) [^{3}H]-IAA transport in decapitated and TIBA, NPA, CHX and lanolin control treated (5 mm above the upper bud) stems from upper bud measured in two stem sections at distance of 0-4 and 4-8 mm under the bud. Statistically significant differences (identified by Student's t-test) $\alpha=0.05$* and $\alpha=0.01$**. Error bars represent standard deviations (n=10).
